# *Walking a tightrope*: A meta‐synthesis from frontline nurses during the COVID‐19 pandemic

**DOI:** 10.1111/nin.12492

**Published:** 2022-04-05

**Authors:** Sara Fernández‐Basanta, Marta Castro‐Rodríguez, María‐Jesús Movilla‐Fernández

**Affiliations:** ^1^ Department of Health Sciences, Faculty of Nursing and Podiatry, Research Group GRINCAR Ferrol Industrial Campus, University of A Coruña Ferrol Spain; ^2^ University Hospital Complex of Ferrol, Galician Health Service (SERGAS) Ferrol Spain

**Keywords:** COVID‐19, Health Services Administration, nursing care, qualitative research

## Abstract

Nursing staff plays a key role in the public health response to the COVID‐19 pandemic, being in the front line of care. This study sought to synthesise the qualitative literature on care experiences of frontline nurses during the COVID‐19 pandemic. A search was conducted on five databases in January 2021. Fifteen qualitative studies met the inclusion criteria and were included in the research, being submitted to interpretive meta‐synthesis according to the eMERGe guide. The final synthesis included a line of argument that shows the experiences of frontline nurses during the COVID‐19 pandemic, divided into three major themes: ‘Instability on the edge of a cliff: unpredictable and unknown context,’ ‘The price of walking the tightrope: the uncertainty surrounding care,’ and ‘Finding the balance to reach the other side: dealing with the emotional demands of care.’ Although essential in the health response to the COVID‐19 pandemic, nurses experienced an emotional impact arising from the hampered care provision. Our results point to need for strengthening the training of nurses and future nurses, creating and promoting measures that contribute to their psycho‐emotional well‐being, ensuring a safe environment for their clinical practice, and promoting their participation in decision‐making processes.

## 1 INTRODUCTION

In December 2019, a cluster of pneumonia of unknown aetiology causing severe respiratory distress syndrome in infected patients was reported to health authorities. Subsequently, these cases were confirmed to be caused by a novel coronavirus called severe acute respiratory syndrome coronavirus 2 (SARS‐COV‐2), giving rise to coronavirus disease (COVID‐19) (Ren et al., 2020; Zhang et al., 2020).

Before the ensuing health crisis, health personnel played a pivotal role as they worked on the COVID‐19 frontline, especially nursing staff (Di Tella et al., 2020; Galehdar et al., 2020). Nurses are a primary care provider, spending the most time with patients and, consequently, at most risk of infection. In a maximum‐difficulty context, such as that imposed by SARS‐COV‐2, the nursing practice faced the uncertainty arising from ignorance surrounding the disease, risk of infection, and the scarcity of resources for treating patients (Galehdar et al., 2021; M. T. González‐Gil, González‐Blázquez, et al., 2021; Qiu et al., 2020). Such circumstances provoke a conflict between the principle of comprehensive care and the personal care of nurses themselves (Bellver Capella, 2020), eventually leading to a series of physical and mental effects due to stress (González‐Rodríguez & Labad, 2020; Luna‐Nemecio, 2020). In extreme cases, these events may result in burnout syndrome, causing professional inefficacy or even the impulse to abandon the profession (Silva‐Gomes & Silva‐Gomes, 2021). Moreover, due to the novelty and relatively little information on COVID‐19, nurses lacked similar previous experiences and, thus, necessary tools to aid in their management of it.

Frontline nurses experienced a massive workload, as well as fatigue, suffering, and frustration from observing patients in their care dying alone and not being able to be cared for in the desired way (M. González‐Gil, Oter‐Quintana, et al., 2021; Shen et al., 2020). They also face anxiety, remorse due to their profession, with lower work efficiency related to fear of transmission (Aksoy & Koçak, 2020). Working in unfamiliar areas was also an additional source of stress for nurses. All these factors resulted in high psychological pressure (Shen et al., 2020). These conditions are associated with mental health problems among healthcare workers, such as a high prevalence of moderate depression, anxiety, and posttraumatic stress disorder (Li et al., 2021). In particular, young age, little work experience, female gender, heavy workload, working in unsafe environments, and lack of training and social support were found to be predictors of posttraumatic stress symptoms (PTSS) (d'Ettorre et al., 2021).

Nurses are fundamental in the treatment and management of patients with COVID‐19, playing an active role in recognising clinical manifestations and patient needs, determining prognostic factors, establishing evidence‐based care practices, and managing nursing issues (Galehdar et al., 2021). In this scenario, investigating their care experiences contributes to improving the care provided, the effectiveness of healthcare teams, and the wellbeing of nurses. Being an issue of global relevance, the most appropriate method for investigating nursing experience is through meta‐synthesis. This method not only provides a new, integrated, and more complete interpretation of findings than the results of individual studies, thus offering a deeper and broader understanding of the investigated theme, but also favours the generalisation of results to other contexts (Bondas et al., 2017).

### 1.1 Aim

The above‐mentioned factors prompted the following question, which guided this meta‐synthesis: What are the care experiences of frontline hospital nurses during the COVID‐19 pandemic? Therefore, this study aimed to synthesise qualitative literature on care experiences of frontline nurses active in the management of patients with COVID‐19.

## 2 METHODS

The adopted methodology consists of a meta‐synthesis, whose aim is to provide a new and integrative interpretation of all qualitative articles examined, contributing much more than individual findings while remaining faithful to the interpretation of each study (Mahtani Chugani et al., 2006). More specifically, this study consisted of a meta‐ethnography (Noblit & Hare, 1988), which consisted of translating study findings into each other and interpreting the results in a synthesis.

### 2.1 Search Methods

In January 2021, an extensive search was conducted in five databases, namely PubMed, Scopus, CINAHL, PsycINFO, and Web of Sciences. The descriptors were defined according to the Medical Subject Headings (MeSH), Descriptors in Health Sciences (DeCS) and the Cumulative Index of Nursing and Allied Health Literature (CINAHL) and combined with the Boolean operators 'AND' and 'OR' (File S1). To ensure a broader search, truncations (*) were also used.

### 2.2 Search Outcomes

Articles had to meet the following criteria to be included in this synthesis: Qualitative or mixed‐method primary articles published in English, Spanish, and Portuguese, aimed to understand the experiences of frontline nurses during the COVID‐19 pandemic in hospital services.

The database search returned 803 articles whereas the manual search returned only one. Of these, 338 articles were removed using the EndNote online software tool for being duplicates. After title and abstract screening, 419 articles were excluded for not meeting the inclusion criteria, leaving 47 articles for full‐text reading. Of these, 15 articles were included in the synthesis. Thirty‐two articles were removed because the phenomenon of interest (*n* = 11), the methodology (*n* = 10), and the sample (*n* = 8) did not meet our inclusion criteria. In addition, 3 were not primary articles. The selection process of this meta‐synthesis is illustrated in a PRISMA flowchart (File S2).

The first author developed the database search, and SFB and MCR conducted the two‐stage screening of results—although all authors were involved in this step, for inclusion and exclusion criteria were refined through it.

### 2.3 Quality appraisal

Two authors independently performed the critical appraisal of each article using the Critical Appraisal Skills Programme (CASP) (Long et al., 2020)—a 10‐question checklist covering aspects related to methodology, objectives, design, results, ethical approval, and applicability. This appraisal is not intended to filter articles, but to provide information to readers. Evaluations results were discussed in group, and articles were considered to be of the highest quality in most of the questions, but the association between researchers and participants was not clearly stated except in one of the articles (File S3).

### 2.4 Data abstraction and synthesis

The first two authors of this study performed a critical reading of the included articles to describe objective, sample, methodology, data collection method, and key results (File S4).

Based on the richest article in terms of data (Lee & Lee, 2020), first‐ and second‐order constructs were extracted from each article (Schütz, 1962) and tabulated in the Microsoft Word along with a brief description of each construct and the line‐by‐line code. The meaning units and codes were discussed within the research group and compared intra‐ and inter‐study for similarities and contrasts through the systematic and sequential comparison of concepts, resulting in the adoption of existing concepts and the formation of new concepts (File S4). This step was conducted by MCR and supervised by SFB and MJMF.

**Figure 1:**
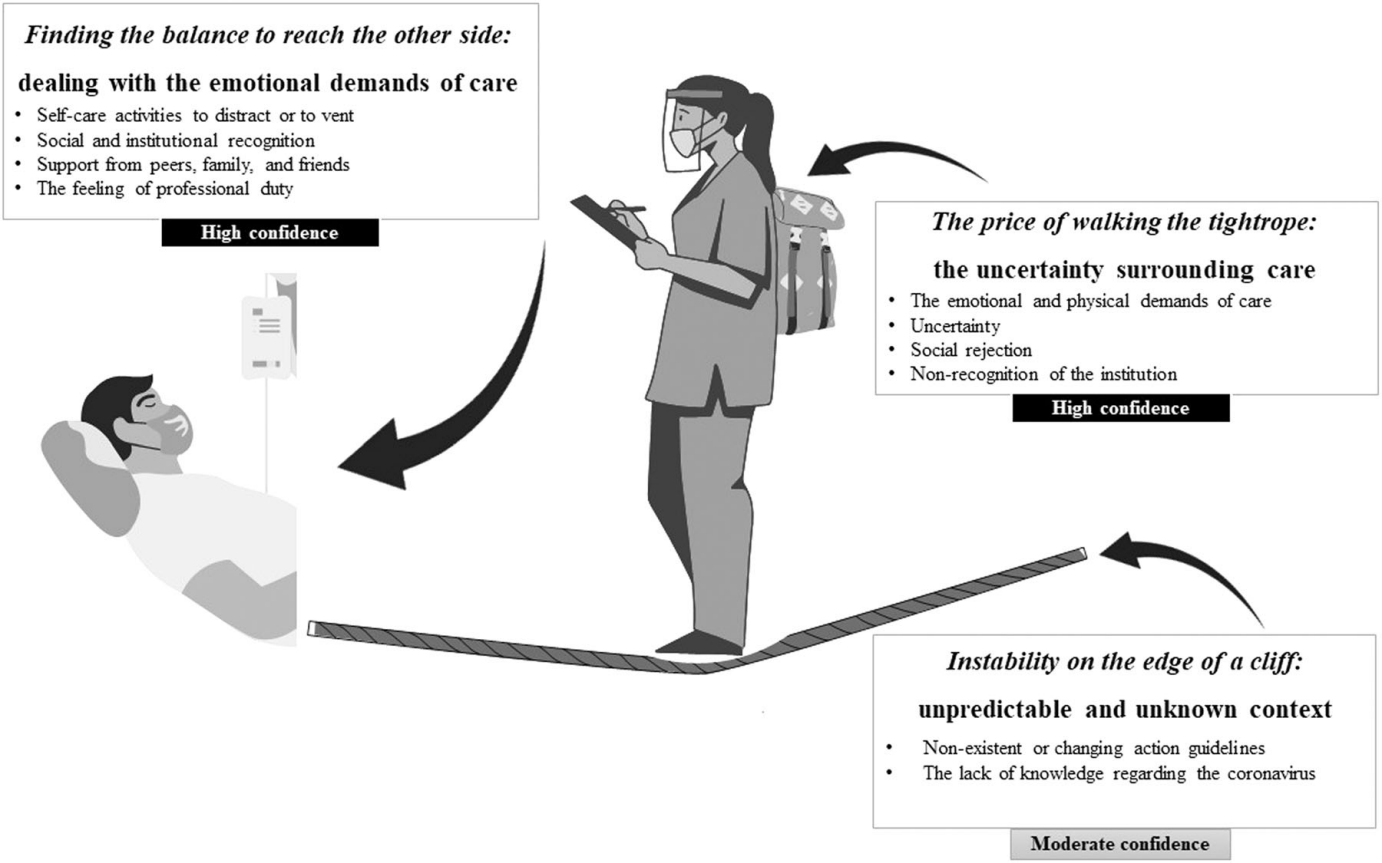
The *Walking the tightrope* lines‐of‐argument synthesis

Translating the studies into one another consisted of comparing the findings from one study with those from another to verify the presence or absence of commonalities. Translation tables allow incorporating the studies findings by reciprocal (concepts in one study can incorporate those of another) and refutational translations (concepts in different studies contradict one another) to form new third‐order concepts (Schütz, 1962). The synthesis process was inductive and involved reflective discussion during the writing of findings. Through an iterative, in‐depth, back‐and‐forth analysis of translations, a lines‐of‐argument synthesis was created based on the metaphorical themes (Figure 1). All authors were involved in these steps.

## 3 RESULTS

This meta‐synthesis was conducted using a sample of qualitative articles, most with descriptive designs (File S2), addressing the experiences of frontline nurses during the COVID‐19 pandemic. These studies were conducted in Spain, Italy, Turkey, Iran, the United States, China, and South Korea, with samples ranging from 10 to 30 participants, resulting in a total of 277. Participants consisted of nurses who cared for patients with COVID‐19 in different types of specialised hospital services, including intensive care units (ICU), emergency services, infectious hospitalisation services, negative pressure rooms, and COVID‐19 units. Data were primarily collected by semi‐structured telephone interviews.

Interpretive findings of the synthesis are presented below. Third‐order constructs (reviewers' interpretation of the translations) are presented with references of the primary articles that contributed to their construction, and the quotations or first‐order constructs (participant quotations).

### 3.1 ‘*Instability on the edge of a cliff*:’ Unpredictable and unknown context

First, the non‐existent or changing action guidelines and the lack of knowledge regarding SARS‐CoV‐2 posed a series of challenges for nurses, which is represented by the unpredictable and unknown context, metaphorically represented through the instability on the edge of a cliff.

Frontline nurses highlighted the changing and often contradictory information, policies, and action procedures against the disease, with most reporting difficulties in maintaining the large amount of information relayed from hospital management. Despite calmly accepting the imposed changes, frontline nurses expressed the ensuing challenge and the effort to be aware of the situation evolution. This synthesis emerged from various studies (Andreu‐Periz et al., 2020; Galehdar et al., 2020; Lee & Lee, 2020; Schroeder et al., 2020).

Whereas, some nurses expressed empathy towards the hospital leadership amid the dynamic situation, acknowledging that ‘they are doing everything they can,’ others were frustrated, especially when the standards did not meet their expectations, as this nurse reported:

‘My experience with that is not that great because the first two weeks COVID came out we had [a hospital administrator] and he just said, 'You don't need any other mask than the drop mask.' Just reinforcing that completely over and over again. The CDC says this. You don't need anything other than the drop mask. He literally saw him scold people. Like take that off, take that out of his pocket. You don't need the N95. He does not have aerosolized patients. It doesn't make sense for you to have that, and then get an email from the same man two weeks later saying, 'Everyone uses N95. Rule out COVID, [Patient under investigation] ‐ whatever ‐ use your N95. '… Why don't we start with all the protection?’ (Schroeder et al., 2020, p. 4)

Before the severity and unpredictability of the disease, as reported by the nurse in the next quote, the lack of protocols in place for nurses caring for infected patients was also a challenging aspect to face, which emerged in other studies (Andreu‐Periz et al., 2020; Galehdar et al., 2020; Kalateh Sadati et al., 2021).

‘Not all COVID‐19 patients have severe clinical symptoms… there is no correlation between a patient's death and clinical symptoms… a patient with mild symptoms may die while another with severe symptoms recovers… the unknown dimensions of the disease are numerous’. (Galehdar et al., 2020, p. 4)

Their concerns were chiefly focused on the degree of transmission and diagnostic complexity, as reported by several studies (Kalateh Sadati et al., 2021; Lee & Lee, 2020). Applying experiential knowledge of other similar infectious diseases to cope with the situation was yet another difficult aspect, especially at the onset of the pandemic. For example, the following nurse lacked sufficient knowledge and skills to manage the COVID‐19 pandemic despite having experience in dealing with the severe acute respiratory syndrome (SARS) virus (Liu et al., 2020):

‘Although I had participated in the fight against SARS in 2003 and the Wenchuan earthquake in 2008, I still felt my skills were inadequate in this epidemic and I hoped to receive systematic disaster relief training’. (Liu et al., 2020, p. 761)

Most nurses of the primary studies had to work in care services where they lacked previous experience, and needed to learn new techniques, procedures, and protocols, as well as to handle equipment they were not familiar with. Moreover, they did not receive specific training to treat patients with infectious diseases. This synthesis was built on several primary studies (Galehdar et al., 2021; Jia et al., 2021; Liu et al., 2020; Tan et al., 2020), and is reflected in this quotation:

‘I do not have the experience of working in the Intensive Care Unit, and I do not know much about the use of the ventilator… Under pressure and on the verge of collapse, we all have to work in high concentrations’ (Tan et al., 2020, p. 1386)

### 3.2 ‘*The price of walking the tightrope*:’ The uncertainty surrounding care

Being at the frontline of patient care in the COVID‐19 pandemic comes at a price, which is represented metaphorically by a backpack. The emotional and physical demands of care, uncertainty, social rejection, and nonrecognition of the institution loaded the nurses' backpack, hindering their balance on the tightrope and precluding their advance to reach the other side.

Nurses feared the fact that they lacked knowledge of the disease and its effects, thus impacting the care provided, which emerged from several studies (Andreu‐Periz et al., 2020; Iheduru‐Anderson, 2021; Liu et al., 2020; Sun et al., 2020). Additionally, nurses also felt that they lacked sufficient clinical experience to cope with the disease and expressed concern on the adequacy of their ability to work in COVID‐19 units. Such feelings occurred primarily before starting a new nursing position, disappearing throughout the days. This interpretation was based primarily on several primary articles (Deliktas Demirci et al., 2021; Galehdar et al., 2020; Schroeder et al., 2020).

A high risk of infection due to close contact with infected patients also emerged in several studies (Catania et al., 2021; Galehdar et al., 2020, 2021; Iheduru‐Anderson, 2021; Karimi et al., 2020). The scarcity of medical resources, especially at the onset, evoked feelings of a lack of protection, leaving healthcare workers vulnerable to SARS‐CoV‐2, as observed in the study by Andreu‐Periz et al. (2020). In fact, being unacquainted with the situation, nurses constantly questioned the safety of personal protective equipment (PPE) and isolation measures, as shown in several studies and in the following statement (Kalateh Sadati et al., 2021; Lee & Lee, 2020; Liu et al., 2020):

‘It was really scary before entering the isolation unit for the first time in personal protective equipment. Even though he had a mask and goggles, will they protect me? Is it safe to breathe?’ (Lee & Lee, 2020, p. 6).

Due to the asymptomatic nature of the disease, the potential of placing their own families at risk led to distress and anxiety, with several studies reporting nurses to either avoiding relatives or even moving their place of residence (Catania et al., 2021; Deliktas Demirci et al., 2021; Galehdar et al., 2020, 2021; Iheduru‐Anderson, 2021; Kalateh Sadati et al., 2021; Karimi et al., 2020; Sun et al., 2020). In this example, the nurse expresses a fear of infecting her family and consequences regarding the care of her children:

‘It was really unprecedented and scary… My mom who was in her mid‐sixties came to take care of my two young children. I wondered as I was leaving if that will be the day, I contract the disease and bring it home to my family, to my mother. What will happen to my children if my mom gets sick and is not able to care for them?’ (Iheduru‐Anderson, 2021, p. 6)

Long working hours, shortages, sick leave of colleagues, and the discomfort of PPE caused frontline nurses to experience physical exhaustion, which was reported by several studies (Andreu‐Periz et al., 2020; Iheduru‐Anderson, 2021; Lee & Lee, 2020; Liu et al., 2020; Tan et al., 2020). Moreover, dealing with the suffering of isolated patients and their families at critical moments, deaths alone, especially of young patients, or the death of other nurses had an emotional impact on participants as reported in different studies (Catania et al., 2021; Iheduru‐Anderson, 2021; Kalateh Sadati et al., 2021; Tan et al., 2020), contributing to burnout, as reflected in this excerpt:

‘I don't think I can go on like this. There is so much death and suffering. I am losing many patients and I am helpless. I have really thought about ending everything so that I don't have to see so much suffering and feel so desperate’ (Iheduru‐Anderson, 2021, p. 8).

Nurses experienced intense frustration with the noncompliance of preventive measures by the general population, which is reported by several studies (Andreu‐Periz et al., 2020; Galehdar et al., 2020). Many nurses reported experiencing a lack of empathy towards and an emotional conflict in caring for patients infected after attending meetings or religious events resulting in outbreaks of SARS‐CoV‐2. Moreover, they often had to deal with uncooperative and non‐empathic patients, as this quotation shows:

‘The government emphasized that there were no mass meetings, and anyone who has been in a mass meeting should get tested… They did not cooperate… Because that's how I saw them, I really couldn't sympathize with them like other patients’ (Lee & Lee, 2020, p. 10)

In several cases, some nurses were even discriminated and rejected by certain individuals under the pretext of being transmitters of the virus. For example, some nurses were unable to use a taxi because they were health workers:

‘Two of our colleagues were going to the hospital. One of them got into a taxi and the driver asked her where she was going. When she told him to take her to the hospital, he asked her to get off her. The same thing happened to another colleague when a driver, after knowing her destination ‐ our hospital ‐ did not allow her to get into the taxi’ (Kalateh Sadati et al., 2021, p. 76)

Along with the lack of recognition and support from hospital management, these factors unleashed the frustration, self‐deprecation, and loss of motivation of nurses to continue on the frontline. Several reports also state that nurses' involvement was rewarded seldomly, and their presence in the decision‐making processes was often disregarded (Deliktas Demirci et al., 2021; Iheduru‐Anderson, 2021; Lee & Lee, 2020).

### 3.3 ‘*Finding the balance to reach the other side*’: Dealing with the emotional demands of care

Finally, in face of this challenging and complex scenario in which nurses were required to deal with the emotional demands of care, they found the balance across the tightrope through self‐care activities to distract or to vent, social and institutional recognition, support from peers, family, and friends, or the feeling of professional duty.

Speculation, isolation, depression, distraction, shyness, irony, and rationalisation were some common responses to the emotional demands of care. Among self‐care activities performed by frontline nurses, meditation and mindfulness, sports or listening to music as a means of distraction, or writing letters or diaries to vent are reported in several studies (Iheduru‐Anderson, 2021; Jia et al., 2021; Kackin et al., 2020; Sun et al., 2020).

Professional recognition on behalf of the institution and society increased the motivation of nurses, with some expressing heartfelt gratitude for the social support received—PPEs, letters, and messages praising their coping efforts, as observed in several primary studies (Deliktas Demirci et al., 2021; Iheduru‐Anderson, 2021; Jia et al., 2021; Lee & Lee, 2020; Liu et al., 2020; Sun et al., 2020). Concurrently, team spirit enabled nurses to provide a safer care and to develop a support network. According to the reports of most of the interviewed nurses, collective power and team cohesion helped them deal with the arising challenges, with a positive environment being indispensable for the maintenance of their work at the frontline (Catania et al., 2021; Deliktas Demirci et al., 2021; Galehdar et al., 2021; Iheduru‐Anderson, 2021; Jia et al., 2021; Kackin et al., 2020; Lee & Lee, 2020; Liu et al., 2020; Sun et al., 2020), as shown in the following quotation:

‘Although we came from different clinics, we all knew that it was a team effort and that we had to support each other. We hug more and work in solidarity. We all learned together. This helped me cope’ (Deliktas Demirci et al., 2021, p. 6)

Despite the risk involved and the ensuing difficulties, most nurses remained on the frontline due to the feeling of professional duty, which was based on the analysis of several studies (Andreu‐Periz et al., 2020; Galehdar et al., 2021; Kalateh Sadati et al., 2021; Liu et al., 2020; Schroeder et al., 2020; Tan et al., 2020). Nurses felt satisfied knowing that they were contributing towards fighting against a global pandemic and even perceived themselves as ‘heroes or soldiers fighting the pandemic’, as shown by the following nurse:

‘There is a war against disease and we fight the pandemic as a soldier on the front line. I believe that this disease will never end if we do not do our duty. It is a very beautiful spiritual satisfaction to be in the pandemic. I realized that we have a very sacred profession as a nurse’ (Deliktas Demirci et al., 2021, p. 5)

### 3.4 *Walking the tightrope*: The lines‐of‐argument synthesis

The lines‐of‐argument synthesis emerged from the reciprocal and refutational translations of the included articles, named under the metaphor *Walking the tightrope* (Figure 1). Determined by three themes, such a metaphor symbolises care experiences during the COVID‐19 pandemic of frontline nurses, who attempt to maintain their balance across the tightrope to provide adequate care for patients. However, certain difficulties cause them to stumble and even fall. According to the metaphor, the unpredictable and unknown context arising from the pandemic is what causes ‘Instability on the edge of a cliff.’ ‘The price of walking the tightrope’ represents the emotional burden caused by caring for patients, characterised by the image of a backpack. Dealing with the emotional demands of care, the support received, and feelings of duty were reasons for engaging in care, depicted through the theme ‘Finding the balance to reach the other side’.

## 4 DISCUSSION

From the analysis of the included articles, the line‐of‐argument synthesis entitled *Walking the tightrope* (Figure 1) was obtained, which depicts the experience of frontline nurses during the unpredictable and unknown context of the COVID‐19 pandemic. Participants reported facing great difficulties imposed by the emotional and physical demands of care, uncertainty, social rejection, and nonrecognition of the institution, which lead to emotional burden, physical exhaustion, and a series of personal conflicts. To continue at the frontline of care, the studied individuals relied on self‐care activities to distract or to vent, social and institutional recognition, the support from peers, family, and friends, or the feeling of professional duty.

Care, and its relational, reciprocal, and transpersonal act between patient and caregiver, is a core element of Nursing (Borré‐Ortiz et al., 2015; Boykin & Schoenhofer, 2010). In this scenario, patient–nurse encounters enable patients to communicate their needs and sufferings, making them bearable (Rehnsfeldt & Eriksson, 2004). According to the Caritative Caring Theory (Eriksson, 1994), the human being is conceived as an entity made up of body, soul, and spirit, so that the provided care must transcend the pathology‐centred biomedical approach.

Within this scope, caring communion means creating possibilities for the other (Lindström et al., 2006) through a mutual understanding in which the nurse seeks to analyse the patient's conceptions of health and disease. In other words, nurses must overcome their vulnerability by engaging with the patient's beliefs, thus allowing for a comprehensive care (Waldow, 2008). Regarding the pandemic context, care provision involved dealing with obstacles that hindered encounters and communion with patients, such as the fear of contagion, poor training, and uncertainty due to a disease with no previous history. Moreover, physical distancing measures to prevent virus transmission, such as the compulsory use of PPEs, could foster a feeling of coldness and distance from the patients, causing an involuntary detachment (Juárez‐Rodríguez & García‐Campos, 2009; Waibel et al., 2012). This is especially important when we consider that patients tend to appreciate continuity of care when they get to know the nursing staff.

Conversely, establishing encounters with the patient, and consequently being exposed to suffering and uncertainties (Thorup et al., 2012), can be configured as an emotional burden for the nurses, who may exposed their own vulnerabilities through care provision (Carel, 2009; Edward & Hercelinskyj, 2007; Gjengedal et al., 2013; Gray, 2009; Hem & Heggen, 2003). Although not often seen as vulnerable, nurses can sometimes expose their vulnerabilities when they get involved in their patients' difficulties (Angel & Vatne, 2017), especially in a scenario of high emotional demand such as that imposed to frontline nurses, who had to face the isolation and lonely death of patients.

In these contexts, nurses are expected to be strong and protect their patients, relying on the ability to manage their own feelings (Thorup et al., 2012)—an unrealistic expectation for nurses who were exposed to situations beyond their control due to lack of knowledge and previous practical experience (Billings et al., 2020; Carel, 2009; Wilkin & Slevin, 2004). Nurses' reports also address the fear of contagion, influenced by knowledge of the disease, information sources, and emotional aspects (Zhong et al., 2021), so that in many cases the perceived risk outweighed the actual risk due to fear, stress, and anxiety. This situation posed a psychological challenge for nurses, inducing mental disorders such as depression, anxiety, or insomnia (Agyei et al., 2021; Billings et al., 2020). Despite the numerous self‐care strategies adopted and the support received by others, nurses remained in the frontline care mostly due to a feeling of professional duty. This finding is aligned with what (Eriksson, 1994) calls *ethos*, the core component of care that refers to the internal duty towards it.

The COVID‐19 pandemic brought diverse uncertainties that rebounded on the care provision by nurses. In this study, we understand uncertainty as a dynamic state in which individuals are unable to assign probabilities to outcomes, thus challenging their sense of self‐confidence or control and causing an uneasiness with repercussions on cognitive, emotional, or behavioural aspects (Koffman et al., 2020; Penrod, 2001). The judgments and decisions of nurses were affected by the uncertainty arising from the insufficient scientific knowledge of the disease and the limitations of empirical knowledge (Thompson & Dowding, 2001). Patients likewise suffered from these uncertainties, seeing themselves alone in an unfamiliar environment and lacking the warmth of human contact.

Our findings highlight that frontline nurses were mentally, emotionally, and physically exhausted with the emotional demands of care and work overload. Such condition presents a risk not only to the care provided, for nurses tend to prioritise the performance of techniques to the detriment of a comprehensive care, but also to health professionals themselves, who may experience a burnout syndrome (Silva‐Gomes & Silva‐Gomes, 2021).

### 4.1 Implications for practice, education and research

The COVID‐19 pandemic has highlighted or aggravated the weaknesses of healthcare systems regarding the working conditions of nursing professionals or the need to strengthen nursing staff (Halcomb et al., 2020). It has exacerbated long and intense daily workloads, arduous workdays, low wages, poor or non‐existent break support structures, and psychological distress (David et al., 2020). The epidemic peaks exacerbate the already‐existing nursing shortage due to the aging population and the nearly 500,000 nurses on the verge of retiring, including nurses' infection with SARS‐CoV‐2 (Crismon et al., 2021; OECD/European Union, 2020). Accordingly, this resulted in many nurses having to provide care in services for which they were not prepared.

In contrast, the pandemic has also shown the invisibility of nurses in areas other than services. Despite the work previously performed and the experiences of such personnel in major epidemics and wars, the absence of nurses in decision‐making during the pandemic, or in the neglect and lack of attention to their well‐being, is evident by institutions or research studies. Therefore, it is imperative to initiate a social dialogue led by nurses, from a critical conscience that encourages and promotes the involvement of institutions. For this, focus should not be placed on what has been learned, but on how as well as and how to reproduce the learning experience. Therefore, strengthening the training of leading nurses is required, as well as a greater social presence, taking advantage of the thrust of the pandemic, which is aware of the potential of nursing as well as the need for its care.

As mentioned previously, the lack of knowledge underpins uncertainty, hence justifying the need for prior training and preparing nurses caring for COVID‐19 patients for the first time, as well as for encouraging students to learn in real‐life clinical settings and in patients affected by the disease, improving training in infectious diseases. Further, resources and spaces that favour the care and self‐care of nurses through training in psychological well‐being or by providing them with psychological support are required. In this context, establishing safe environments for care provision for both patients and nurses and incorporating nurses into decision‐making processes are essential.

Consequently, studies investigating the experience of nurses in other clinical settings highly affected by the pandemic, such as primary care or geriatrics, are essential. This will facilitate knowledge of the care needs of the particular group under care and establishing a starting point for the involvement of the organisation in favour of nursing.

### 4.2 Strengths and limitations

The adoption of the meta‐ethnography method (Noblit & Hare, 1988) enabled us to group and synthesise results from numerous studies, generating interpretive findings that facilitate a broader perspective of the experiences of frontline nurses during the COVID‐19 pandemic. This study performed an exhaustive search for studies conducted in different geographical contexts, including those written in English, Spanish, and Portuguese—although only articles in English and one in Spanish assembled our study sample.

This meta‐synthesis was elaborated according to the eMERGe guide (France et al., 2019), improving the transparency and integrity of our research process (File S5), and the articles were critically appraised with the CASP tool (Long et al., 2020).

This study has limitations as to its timeframe, for more articles on the theme may have been published since its performance in January 2021. However, the overall sample provided a valuable and highly reliable picture of the acute phase of the pandemic. In contrast, the meta‐synthesis collects the experiences of nurses working in different types of specialised hospital services, with no other services or clinical settings represented, such as primary care or nursing homes.

## 5 CONCLUSION

This meta‐synthesis provides a new and broader interpretation to a topic of global relevance: the experience of frontline nurses during the COVID‐19 pandemic. Providing care in an unpredictable context, in which new knowledge emerges daily, frontline nurses are constantly faced with the emotional and physical demands of care, uncertainty, social rejection, and nonrecognition. With that, these professionals experienced emotional burden, physical exhaustion, and personal conflicts regarding care and patients' perception, remaining in the frontline of care due to self‐care activities, social and institutional recognition, feeling of duty, and the support of peers, family, and friends.

This study implications are particularly valuable for the practice and education of nurses, health professionals, and managers, suggesting the need for strengthening the training of nurses and future nurses regarding this new disease, creating and promoting resources that contribute to their psycho‐emotional well‐being, ensuring safe environments for their performance, and incorporating them into decision‐making processes.

## CONFLICTS OF INTEREST

The authors declare no conflicts of interest.

## Supporting information

Supporting information.

Supporting information.

Supporting information.

Supporting information.

Supporting information.

## Data Availability

The data supporting the findings of this study are available on request from the corresponding author.

## References

[nin12492-bib-0001] Agyei, F. , Bayuo, J. , Baffour, P. , & Laari, C. (2021). ‘Surviving to thriving’: A meta‐ethnography of the experiences of healthcare staff caring for persons with COVID‐19. BMC Health Services Research, 21(1), 1–14. 10.1186/s12913-021-07112-w 34670562 PMC8528651

[nin12492-bib-0002] Aksoy, Y. E. , & Koçak, V. (2020). Psychological effects of nurses and midwives due to COVID‐19 outbreak: The case of Turkey. Archives of Psychiatric Nursing, 34(5), 427–433. 10.1016/j.apnu.2020.07.011. 33032769 PMC7341051

[nin12492-bib-0003] Andreu‐Periz, D. , Ochando‐García, A. , & Limón‐Cáceres, E. (2020). Experiencias de vida y soporte percibido por las enfermeras de las unidades de hemodiálisis hospitalaria durante la pandemia de COVID‐19 en España [Life experiences and support perceived by nurses in hospital haemodialysis units during the COVID‐19 pandemic in Spain]. Enfermería Nefrológica, 23(2), 148–159. 10.37551/s2254-28842020022

[nin12492-bib-0004] Angel, S. , & Vatne, S. (2017). Vulnerability in patients and nurses and the mutual vulnerability in the patient–nurse relationship. Journal of Clinical Nursing, 26(9–10), 1428–1437. 10.1111/jocn.13583 27626897

[nin12492-bib-0005] Bellver Capella, V. (2020). Problemas bioéticos en la prestación de los cuidados enfermeros durante la pandemia del COVID‐19 [Bioethical problems in the provision of nursing care during the COVID‐19 pandemic]. Index de Enfermería, 29(1–2), 46–50.

[nin12492-bib-0006] Billings, J. , Ching, B. C. F. , Gkofa, V. , Greene, T. , & Bloomfield, M. (2020). Healthcare workers experiences of working on the frontline and views about support during COVID‐19 and comparable pandemics: A rapid review and meta‐synthesis. MedRxiv, 2020.06.21 20136705. 10.1101/2020.06.21.20136705 PMC8419805 34488733

[nin12492-bib-0007] Bondas, T. , Hall, E. , & Wikberg, A. (2017). Metasynthesis in health care research, Research methods in health (pp. 325–342). Oxford University Press.

[nin12492-bib-0008] Borré‐Ortiz, Y. M. , Lenis‐Victoria, C. , Suárez‐Villa, M. , & Tafur‐Castillo, J. (2015). El conocimiento disciplinar en el currículo de enfermería: Una necesidad vital para transformar la práctica [Disciplinary knowledge in nursing curriculum: A vital need to transform practice]. Revista Ciencias de la Salud, 13(3), 481–491. 10.12804/revsalud13.03.2015.12

[nin12492-bib-0009] Boykin, A. , & Schoenhofer, S. O. (2010). Anne Boykin and Savina O. Schoenhofer's nursing as caring theory. In M. E. Parker (Ed.), Nursing theories and nursing practice (2nd ed., pp. 370–385). F.A. Davis Company.

[nin12492-bib-0010] Carel, H. (2009). A reply to ‘Towards an understanding of nursing as a response to human vulnerability by Derek Sellman: Vulnerability and illness. Nursing Philosophy, 10(3), 214–219. 19527442 10.1111/j.1466-769X.2009.00401.x

[nin12492-bib-0011] Catania, G. , Zanini, M. , Hayter, M. , Timmins, F. , Dasso, N. , Ottonello, G. , Aleo, G. , Sasso, L. , & Bagnasco, A. (2021). Lessons from Italian front‐line nurses' experiences during the COVID‐19 pandemic: A qualitative descriptive study. Journal of Nursing Management, 29(3), 404–411. 10.1111/jonm.13194 33107657

[nin12492-bib-0012] Crismon, D. , Mansfield, K. J. , Hiatt, S. O. , Christensen, S. S. , & Cloyes, K. G. (2021). COVID‐19 pandemic impact on experiences and perceptions of nurse graduates. Journal of Professional Nursing, 37(5), 857–865. 10.1016/j.profnurs.2021.06.008 34742515 PMC9767315

[nin12492-bib-0013] David, H. M. S. L. , Acioli, S. , Silva, M. R. F. d , Bonetti, O. P. , & Passos, H. (2020). Pandemics, crisis conjunctures, and professional practices: What is the role of nursing with regard to COVID‐19? Revista Gaúcha de Enfermagem, 42. 10.1590/1983-1447.2021.20190254 33084792

[nin12492-bib-0014] Deliktas Demirci, A. , Oruc, M. , & Kabukcuoglu, K. (2021). ‘It was difficult, but our struggle to touch lives gave us strength’: The experience of nurses working on COVID‐19 wards. Journal of Clinical Nursing, 30(5‐6), 732–741. 10.1111/jocn.15602 33325080

[nin12492-bib-0015] d'Ettorre, G. , Ceccarelli, G. , Santinelli, L. , Vassalini, P. , Innocenti, G. P. , Alessandri, F. , Koukopolous, A. E. , Russo, A. , d'Ettore, G. , & Tarsitani, L. (2021). Post‐traumatic stress symptoms in healthcare workers dealing with the COVID‐19 pandemic: A systematic review. International Journal of Environmental Research and Public Health, 18(2), 601. 10.3390/ijerph18020601 PMC7828167 33445712

[nin12492-bib-0016] Di Tella, M. , Romeo, A. , Benfante, A. , & Castelli, L. (2020). Mental health of healthcare workers during the COVID‐19 pandemic in Italy. Journal of Evaluation in Clinical Practice, 26(6), 1583–1587. 10.1111/jep.13444 32710481

[nin12492-bib-0017] Edward, K.‐l , & Hercelinskyj, G. (2007). Burnout in the caring nurse: Learning resilient behaviours. British Journal of Nursing, 16(4), 240–242. 10.12968/bjon.2007.16.4.22987 17363857

[nin12492-bib-0018] Eriksson, K. (1994). Theories of caring as health. In D. A. Gaut , & A. Boykin (Eds.), Caring as healing: Renewal through hope (pp. 3–20). Jones & Bartlett Learning.

[nin12492-bib-0019] France, E. F. , Cunningham, M. , Ring, N. , Uny, I. , Duncan, E. , Jepson, R. G. , Maxwell, M. , Roberts, R. J. , Turley, R. L. , Booth, A. , Britten, N. , Flemming, K. , Gallagher, I. , Garside, R. , Hannes, K. , Lewin, S. , Noblit, G. W. , Pope, C. , Thomas, J. , … Noyes, J. (2019). Improving reporting of meta‐ethnography: The eMERGe reporting guidance. BMC Medical Research Methodology, 19(1), 25. 10.1186/s12874-018-0600-0 30709371 PMC6359764

[nin12492-bib-0020] Galehdar, N. , Kamran, A. , Toulabi, T. , & Heydari, H. (2020). Exploring nurses' experiences of psychological distress during care of patients with COVID‐19: A qualitative study. BMC Psychiatry, 20(1), 1–9. 10.1186/s12888-020-02898-1 33023535 PMC7538040

[nin12492-bib-0021] Galehdar, N. , Toulabi, T. , Kamran, A. , & Heydari, H. (2021). Exploring nurses' perception of taking care of patients with coronavirus disease (COVID‐19): A qualitative study. Nursing Open, 8(1), 171–179. 10.1002/nop2.616 33318825 PMC7729793

[nin12492-bib-0022] Gjengedal, E. , Ekra, E. M. , Hol, H. , Kjelsvik, M. , Lykkeslet, E. , Michaelsen, R. , Orøy, A. , Skrondal, T. , Sundal, H. , Vatne, S. , & Wogn‐Henriksen, K. (2013). Vulnerability in health care: Reflections on encounters in every day practice. Nursing Philosophy, 14(2), 127–138. 10.1111/j.1466-769X.2012.00558.x 23480039

[nin12492-bib-0023] González‐Gil, M. , Oter‐Quintana, C. , Martínez‐Marcos, M. , Alcolea‐Cosín, M. , Navarta‐Sánchez, M. , Robledo‐Martín, J. , Palmar‐Santos, A. , Pedraz‐Marcos, A. , González‐Blázquez, C. , Parro‐Moreno, A. I. , & Otero‐García, L. (2021). El valor del recurso humano: Experiencia de profesionales enfermeros de cuidados críticos durante la pandemia por COVID‐19 [The value of human resources: Experience of critical care nurses during the COVID‐19 epidemic]. Enfermería Intensiva. 10.1016/j.enfi.2021.09.005 PMC8637435 34873389

[nin12492-bib-0024] González‐Gil, M. T. , González‐Blázquez, C. , Parro‐Moreno, A. I. , Pedraz‐Marcos, A. , Palmar‐Santos, A. , Otero‐García, L. , Navarta‐Sánchez, M. V. , Alcolea‐Cosín, M. T. , Argüello‐López, M. T. , Canalejas‐Pérez, C. , Carrillo‐Camacho, M. E. , Casillas‐Santana, M. L. , Díaz‐Martínez, M. L. , García‐González, A. , García‐Perea, E. , Martínez‐Marcos, M. , Martínez‐Martín, M. L. , Palazuelos‐Puerta, M. , Sellán‐Soto, C. , & Oter‐Quintana, C. (2021). Nurses' perceptions and demands regarding COVID‐19 care delivery in critical care units and hospital emergency services. Intensive and Critical Care Nursing, 62, 102966. 10.1016/j.iccn.2020.102966 33172732 PMC7598734

[nin12492-bib-0025] González‐Rodríguez, A. , & Labad, J. (2020). Salud mental en tiempos de la COVID: Reflexiones tras el estado de alarma [Mental health in times of COVID: Thoughts after the state of alarm]. Medicina Clínica, 155(9), 392–394. 10.1016/j.medcli.2020.07.009 PMC7381887 32958264

[nin12492-bib-0026] Gray, B. (2009). The emotional labour of nursing: Defining and managing emotions in nursing work. Nurse Education Today, 29(2), 168–175. 10.1016/j.nedt.2008.08.003 18793817

[nin12492-bib-0027] Halcomb, E. , McInnes, S. , Williams, A. , Ashley, C. , James, S. , Fernandez, R. , Stephen, C. , & Calma, K. (2020). The experiences of primary healthcare nurses during the COVID‐19 pandemic in Australia. Journal of Nursing Scholarship, 52(5), 553–563. 10.1111/jnu.12589 32735758 PMC7436753

[nin12492-bib-0028] Hem, M. H. , & Heggen, K. (2003). Being professional and being human: One nurse's relationship with a psychiatric patient. Journal of Advanced Nursing, 43(1), 101–108. 10.1046/j.1365-2648.2003.02677.x 12801401

[nin12492-bib-0029] Iheduru‐Anderson, K. (2021). Reflections on the lived experience of working with limited personal protective equipment during the COVID‐19 crisis. Nursing Inquiry, 28(1), e12382. 10.1111/nin.12382 33010197 PMC7646033

[nin12492-bib-0030] Jia, Y. , Chen, O. , Xiao, Z. , Xiao, J. , Bian, J. , & Jia, H. (2021). Nurses' ethical challenges caring for people with COVID‐19: A qualitative study. Nursing Ethics, 28(1), 33–45. 10.1177/0969733020944453 32856534 PMC7653013

[nin12492-bib-0031] Juárez‐Rodríguez, P. A. , & García‐Campos, M. dL. (2009). La importancia del cuidado de enfermería [The importance of nursing care]. Revista de Enfermería del Instituto Mexicano del Seguro Social, 17(2), 113–115.

[nin12492-bib-0032] Kackin, O. , Ciydem, E. , Aci, O. S. , & Kutlu, F. Y. (2020). Experiences and psychosocial problems of nurses caring for patients diagnosed with COVID‐19 in Turkey: A qualitative study. International Journal of Social Psychiatry, 67(2), 158–167. 10.1177/0020764020942788. 32674644

[nin12492-bib-0033] Kalateh Sadati, A. , Zarei, L. , Shahabi, S. , Heydari, S. T. , Taheri, V. , Jiriaei, R. , Ebrahimzade, N. , & Lankarani, K. B. (2021). Nursing experiences of COVID‐19 outbreak in Iran: A qualitative study. Nursing Open, 8(1), 72–79. 10.1002/nop2.604 32904939 PMC7461197

[nin12492-bib-0034] Karimi, Z. , Fereidouni, Z. , Behnammoghadam, M. , Alimohammadi, N. , Mousavizadeh, A. , Salehi, T. , Mirzaee, M. S. , & Mirzaee, S. (2020). The lived experience of nurses caring for patients with COVID‐19 in Iran: A phenomenological study. Risk Management and Healthcare Policy, 13, 1271–1278. 10.2147/RMHP.S258785 32904130 PMC7450521

[nin12492-bib-0035] Koffman, J. , Gross, J. , Etkind, S. N. , & Selman, L. (2020). Uncertainty and COVID‐19: How are we to respond? Journal of the Royal Society of Medicine, 113(6), 211–216. 10.1177/0141076820930665 32521198 PMC7439590

[nin12492-bib-0036] Lee, N. , & Lee, H.‐J. (2020). South Korean nurses' experiences with patient care at a COVID‐19 designated hospital: Growth after the frontline battle against an infectious disease pandemic. International Journal of Environmental Research and Public Health, 17(23), 9015. 10.3390/ijerph17239015 PMC7729510 33287343

[nin12492-bib-0037] Li, Y. , Scherer, N. , Felix, L. , & Kuper, H. (2021). Prevalence of depression, anxiety and post‐traumatic stress disorder in health care workers during the COVID‐19 pandemic: A systematic review and meta‐analysis. PLoS One, 16(3), e0246454. 10.1371/journal.pone.0246454 33690641 PMC7946321

[nin12492-bib-0038] Lindström, U. , Lindholm, L. , & Zetterlund, J. E. (2006). Theory of caritative caring. In M. A. Alligood (Ed.), Nursing theorists and their work (pp. 191–223). Elsevier.

[nin12492-bib-0039] Liu, Y. E. , Zhai, Z. C. , Han, Y. H. , Liu, Y. L. , Liu, F. P. , & Hu, D. Y. (2020). Experiences of front‐line nurses combating coronavirus disease‐2019 in China: A qualitative analysis. Public Health Nursing, 37(5), 757–763. 10.1111/phn.12768 32677072 PMC7405388

[nin12492-bib-0040] Long, H. A. , French, D. P. , & Brooks, J. M. (2020). Optimising the value of the critical appraisal skills programme (CASP) tool for quality appraisal in qualitative evidence synthesis. Research Methods in Medicine & Health Sciences, 1(1), 31–42. 10.1177/2632084320947559

[nin12492-bib-0041] Luna‐Nemecio, J. (2020). Determinaciones socioambientales del COVID‐19 y vulnerabilidad económica, espacial y sanitario‐institucional [Socio‐environmental determinations of COVID‐19 and economic, spatial and health‐institutional vulnerability]. Revista de Ciencias Sociales (Ve), 26(2), 21–26.

[nin12492-bib-0042] Mahtani Chugani, V. , Axpe Caballero, M. , Serrano Aguilar, P. , González Castro, I. , & Fernández Vega, E. (2006). Metodología para incorporar los estudios cualitativos en la evaluación de tecnologías sanitarias [Methodology to incorporate qualitative studies in the evaluation of health technologies]. Plan Nacional para el SNS del MSC. Servicio de Evaluación del Servicio Canario de Salud.

[nin12492-bib-0043] Noblit, G. W. , & Hare, R. D. (1988). Meta‐ethnography: Synthesizing qualitative studies. Sage Publications.

[nin12492-bib-0044] OECD/European Union . (2020). Health at a Glance: Europe 2020, State of Health in the EU Cycle. OECD Publishing. 10.1787/82129230-en

[nin12492-bib-0045] Penrod, J. (2001). Refinement of the concept of uncertainty. Journal of Advanced Nursing, 34(2), 238–245. 10.1046/j.1365-2648.2001.01750.x 11430286

[nin12492-bib-0046] Qiu, H. , Tong, Z. , Ma, P. , Hu, M. , Peng, Z. , Wu, W. , & Du, B. (2020). Intensive care during the coronavirus epidemic. Intensive Care Medicine, 46, 576–578. 10.1007/s00134-020-05966-y 32077996 PMC7080064

[nin12492-bib-0047] Rehnsfeldt, A. , & Eriksson, K. (2004). The progression of suffering implies alleviated suffering. Scandinavian Journal of Caring Sciences, 18(3), 264–272. 10.1111/j.1471-6712.2004.00281.x 15355520

[nin12492-bib-0048] Ren, L. L. , Wang, Y. M. , Wu, Z. Q. , Xiang, Z. C. , Guo, L. , Xu, T. , Jiang, Y. Z. , Xiong, Y. , Li, Y. J. , Li, X. W. , Li, H. , Fan, G. H. , Gu, X. Y. , Xiao, Y. , Gao, H. , Xu, J. Y. , Yang, F. , Wang, X. M. , Wu, C. , … Wang, J. W. (2020). Identification of a novel coronavirus causing severe pneumonia in human: A descriptive study. Chinese Medical Journal, 133(09), 1015–1024. 10.1097/CM9.0000000000000722 32004165 PMC7147275

[nin12492-bib-0049] Schroeder, K. , Norful, A. A. , Travers, J. , & Aliyu, S. (2020). Nursing perspectives on care delivery during the early stages of the covid‐19 pandemic: A qualitative study. International Journal of Nursing Studies Advances, 2, 100006. 10.1016/j.ijnsa.2020.100006 32864632 PMC7446648

[nin12492-bib-0050] Schütz, A. (1962). Collected papers (1). Martinus Nijhoff.

[nin12492-bib-0051] Shen, X. , Zou, X. , Zhong, X. , Yan, J. , & Li, L. (2020). Psychological stress of ICU nurses in the time of COVID‐19. Critical Care, 24(1), 1–3. 10.1186/s13054-020-02926-2 32375848 PMC7202793

[nin12492-bib-0052] Silva‐Gomes, R. N. , & Silva‐Gomes, V. T. (2021). COVID‐19 pandemic: Burnout syndrome in healthcare professionals working in field hospitals in Brazil. Enfermería Clínica [English Edition], 31(2), 128–129. 10.1016/j.enfcle.2020.10.002 PMC7603962 38620439

[nin12492-bib-0053] Sun, N. , Wei, L. , Shi, S. , Jiao, D. , Song, R. , Ma, L. , Wang, H. , Wang, C. , Wang, Z. , You, Y. , Liu, S. , & Wang, H. (2020). A qualitative study on the psychological experience of caregivers of COVID‐19 patients. American Journal of Infection Control, 48(6), 592–598. 10.1016/j.ajic.2020.03.018 32334904 PMC7141468

[nin12492-bib-0054] Tan, R. , Yu, T. , Luo, K. , Teng, F. , Liu, Y. , Luo, J. , & Hu, D. (2020). Experiences of clinical first‐line nurses treating patients with COVID‐19: A qualitative study. Journal of Nursing Management, 28(6), 1381–1390. 10.1111/jonm.13095 32657465 PMC7404505

[nin12492-bib-0055] Thompson, C. , & Dowding, D. (2001). Responding to uncertainty in nursing practice. International Journal of Nursing Studies, 38(5), 609–615. 10.1016/S0020-7489(00)00103-6 11524107

[nin12492-bib-0056] Thorup, C. B. , Rundqvist, E. , Roberts, C. , & Delmar, C. (2012). Care as a matter of courage: Vulnerability, suffering and ethical formation in nursing care. Scandinavian Journal of Caring Sciences, 26(3), 427–435. 10.1111/j.1471-6712.2011.00944.x 22070455

[nin12492-bib-0057] Waibel, S. , Henao, D. , Aller, M.‐B. , Vargas, I. , & Vázquez, M.‐L. (2012). What do we know about patients' perceptions of continuity of care? A meta‐synthesis of qualitative studies. International Journal for Quality in Health Care, 24(1), 39–48. 10.1093/intqhc/mzr068 22146566

[nin12492-bib-0058] WaldowV, B. R. (2008). El processo de cuidar según la perspectiva de la vulnerabilidad [The caregiving process in the vulnerability perspective]. Revista Latino‐Americana de Enfermagem, 16(4), 765–771. 10.1590/S0104-11692008000400018 18833461

[nin12492-bib-0059] Wilkin, K. , & Slevin, E. (2004). The meaning of caring to nurses: an investigation into the nature of caring work in an intensive care unit. Journal of Clinical Nursing, 13(1), 50–59. 10.1111/j.1365-2702.2004.00814.x 14687293

[nin12492-bib-0060] Zhang, T. , Wu, Q. , & Zhang, Z. (2020). Probable pangolin origin of SARS‐CoV‐2 associated with the COVID‐19 outbreak. Current Biology, 30(7), 1346–1351.e1342. 10.1016/j.cub.2020.03.022 32197085 PMC7156161

[nin12492-bib-0061] Zhong, Y. , Liu, W. , Lee, T.‐Y. , Zhao, H. , & Ji, J. (2021). Risk perception, knowledge, information sources and emotional states among COVID‐19 patients in Wuhan, China. Nursing Outlook, 69(1), 13–21. 10.1016/j.outlook.2020.08.005 32980153 PMC7442898

