# Supplementary material for: Walking a tightrope: A meta‐synthesis from frontline nurses during the COVID‐19 pandemic
Source: Nurs Inq. 2022 Apr 5:e12492. Online ahead of print. doi: 10.1111/nin.12492 (PMC9115365; doi:10.1111/nin.12492)
Supplement: Supplementary file 1 — Supporting information. [file NIN-9999-0-s004.docx]

**Supplementary File 1** Search strategy

| 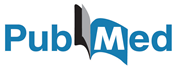 | Pubmed |
| --- | --- |

| S1 | "experienc*" [tw] | 1,157,027 |
| --- | --- | --- |
| S2 | "view*" [tw] | 483,355 |
| S3 | "opinion*" [tw] | 122,468 |
| S4 | "Attitud*"[tw] | 434,414 |
| S5 | "Emotions"[Mesh] | 250,916 |
| S6 | "Knowledge"[Mesh] | 11,662 |
| S7 | "need*" [tw] | 1,964,225 |
| S8 | "Adaptation, Psychological"[Mesh] | 128,997 |
| S9 | OR S1-S8 | 3,875,516 |
| ((((((("experienc*" [tw]) OR ("view*" [tw])) OR ("opinion*" [tw])) OR ("Attitud*"[tw])) OR ("Emotions"[Mesh])) OR ("Knowledge"[Mesh])) OR ("need*" [tw])) OR ("Adaptation, Psychological"[Mesh]) | | |
| S10 | "Nurses"[Mesh] | 89,264 |
| S11 | "nurs*" [Title/Abstract] | 471,463 |
| S12 | caring [tw] | 42,762 |
| S13 | OR S10-S12 | 534,205 |
| (("Nurses"[Mesh]) OR ("nurs*" [Title/Abstract])) OR (caring [tw]) | | |
| S14 | "COVID-19" [tw] | 80,632 |
| S15 | "coronavirus outbreak*" [tw] | 464 |
| S16 | "COVID-19 Pandemic" [tw] | 23,387 |
| S17 | "Coronavirus disease" [tw] | 20,48 |
| S18 | OR S14-S17 | 81,891 |
| ((("COVID-19" [tw]) OR ("coronavirus outbreak*" [tw])) OR ("COVID-19 Pandemic" [tw])) OR ("Coronavirus disease" [tw]) | | |
| S19 | "Qualitative Research"[Mesh] | 59,361 |
| S20 | "qualitative" [tw] | 259,596 |
| S21 | “phenomenology” [tw] | 27,571 |
| S22 | “grounded theory” [tw] | 12,269 |
| S23 | “ethnograph*” [tw] | 11,394 |
| S24 | “interview*” [tw] | 403,095 |
| S25 | "Narration"[Mesh] | 8,701 |
| S26 | "narrative*" [tw] | 51,67 |
| S27 | S19 OR S26 | 633,613 |
| ("experienc*" [tw] OR "view*" [tw] OR "opinion*" [tw] OR "Attitud*"[tw] OR "Emotions"[Mesh] OR "Knowledge"[Mesh] OR "need*" [tw] OR "Adaptation, Psychological"[Mesh]) AND ("Nurses"[Mesh] OR "nurs*" [Title/Abstract] OR caring [tw]) AND ("COVID-19" [tw] OR "coronavirus outbreak*" [tw] OR "COVID-19 Pandemic" [tw] OR "Coronavirus disease" [tw]) AND ("Qualitative Research" [Mesh] OR "qualitative" [tw] OR "phenomenolog*" [tw] OR "grounded theory" [tw] OR "ethnograph*" [tw] OR "interview*" [tw] OR "Narration"[Mesh] OR "narrative*" [tw]) | | |
| Language: English, Portuguese and Spanish | | |
| TOTAL: 156 | | |

| 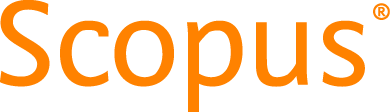 | Scopus |
| --- | --- |

| S1 | TITLE-ABS-KEY(“experienc*”) | 2,578,402 |
| --- | --- | --- |
| S2 | TITLE-ABS-KEY (“view*”) | 1,388,490 |
| S3 | TITLE-ABS-KEY (“opinion*”) | 301,519 |
| S4 | TITLE-ABS-KEY ("Attitude*") | 900,887 |
| S5 | TITLE-ABS-KEY ("Emotion*") | 500,215 |
| S6 | TITLE-ABS-KEY ("Know*") | 5,387,017 |
| S7 | TITLE-ABS-KEY (“need*”) | 4,748,367 |
| S8 | TITLE-ABS-KEY ("cope") | 140,022 |
| S9 | TITLE-ABS-KEY ( " coping " ) | 137,317 |
| S10 | TITLE-ABS-KEY ( " feeling*" ) | 152,514 |
| S11 | OR S1-S10 | 13,754,561 |
| (TITLE-ABS-KEY (“experienc*” OR “view*” OR “opinion*” OR "Attitude*" OR "Emotion*" OR "Know*" OR “need*” OR "cope" OR coping OR feeling*)) | | |
| S12 | TITLE-ABS-KEY ("Nurs*") | 885,940 |
| S13 | TITLE-ABS-KEY ("first-line nurse*") | 95 |
| S14 | TITLE-ABS-KEY ("nursing care") | 64,032 |
| S15 | OR S12-S14 | 885,941 |
| (TITLE-ABS-KEY ("Nurs*" OR "first-line nurse*" OR "nursing care")) | | |
| S16 | TITLE-ABS-KEY ("COVID-19") | 84,429 |
| S17 | TITLE-ABS-KEY ("Coronavirus") | 90,619 |
| S18 | TITLE-ABS-KEY ("coronavirus outbreak*") | 587 |
| S19 | TITLE-ABS-KEY ("COVID-19 Pandemic") | 26,632 |
| S20 | TITLE-ABS-KEY (“Coronavirus disease”) | 54,010 |
| S21 | OR S216-S20 | 115,825 |
| (TITLE-ABS-KEY ("COVID-19" OR "Coronavirus" OR "coronavirus outbreak*" OR "COVID-19 Pandemic" OR “Coronavirus disease”)) | | |
| S22 | TITLE-ABS-KEY ( "Qualitative Research" ) | 127,864 |
| S23 | TITLE-ABS-KEY ( "qualitative" ) | 733,105 |
| S24 | TITLE-ABS-KEY ( "phenomenology" ) | 142,583 |
| S25 | TITLE-ABS-KEY ( "grounded theory" ) | 25,701 |
| S26 | TITLE-ABS-KEY ( "ethnography" ) | 81,624 |
| S27 | TITLE-ABS-KEY ( "interview" ) | 803,136 |
| S28 | TITLE-ABS-KEY ( "focus group" ) | 95,361 |
| S29 | TITLE-ABS-KEY ( "Narration" ) | 17,642 |
| S30 | TITLE-ABS-KEY ( "Narrative*" ) | 201,400 |
| S31 | S22 OR S30 | 1,745,057 |
| (TITLE-ABS-KEY ("Qualitative Research" OR qualitative OR phenomenolog* OR "grounded theory" OR ethnograph* OR interview* OR "focus group*" OR narration OR narrative*)) | | |
| (TITLE-ABS-KEY (“experienc*” OR “view*” OR “opinion*” OR "Attitude*" OR "Emotion*" OR "Know*" OR “need*” OR "cope" OR coping OR feeling*))  AND (TITLE-ABS-KEY ("Nurs*" OR "first-line nurse*" OR "nursing care")) AND  (TITLE-ABS-KEY ("COVID-19" OR "Coronavirus" OR "coronavirus outbreak*" OR "COVID-19 Pandemic" OR “Coronavirus disease”)) AND (TITLE-ABS-KEY ("Qualitative Research" OR qualitative OR phenomenolog* OR "grounded theory" OR ethnograph* OR interview* OR "focus group*" OR narration OR narrative*)) AND ( LIMIT-TO ( LANGUAGE , "English " ) OR LIMIT-TO ( LANGUAGE , "Portuguese " ) OR LIMIT-TO ( LANGUAGE , "Spanish " ) ) | | |
| Language: English, Portuguese and Spanish. | | |
| TOTAL: 172 | | |

| 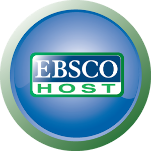 | Cinahl |
| --- | --- |

| S1 | TX: ( experienc* OR view* OR opinion* OR (MH "Nurse Attitudes") OR perspective* OR belief* OR (MH "Emotions") OR need* OR (MH "Coping") |
| --- | --- |
| S2 | TX: ( (MM “Nurses”) OR (MH “Nursing Care”) OR “car*” OR “nurs*”) |
| S3 | TX ( (MH "COVID-19") OR (MH "Coronavirus Infections") OR "coronavirus outbreak*" OR "COVID-19 Pandemic" OR “Coronavirus disease” ) |
| S4 | TX ( (MH”Qualitative Studies+”) OR “qualitative” OR (MH “Interviews+”) OR (MM “Focus groups”) OR (MH “Narratives”) |
| S5 | S1 AND S2 |
| S6 | S3 AND S4 |
| S7 | S5 AND S6 |
|  | **TX ( experienc* OR view* OR opinion* OR (MH "Nurse Attitudes") OR perspective* OR belief* OR (MH "Emotions") OR need* OR (MH "Coping") ) AND TX ( (MM "Nurses") OR (MH "Nursing Care") OR "car*" OR “nurs*” ) AND TX ( (MH "COVID- 19") OR (MH "Coronavirus Infections") OR "coronavirus outbreak*" OR "COVID- 19 Pandemic" OR “Coronavirus disease” ) AND TX ( (MH "Qualitative Studies+") OR "qualitative" OR (MH "Interviews+") OR (MM "Focus Groups") OR (MH "Narratives") )** |
| Language: English, Portuguese and Spanish. | |
| TOTAL: 191 | |

| 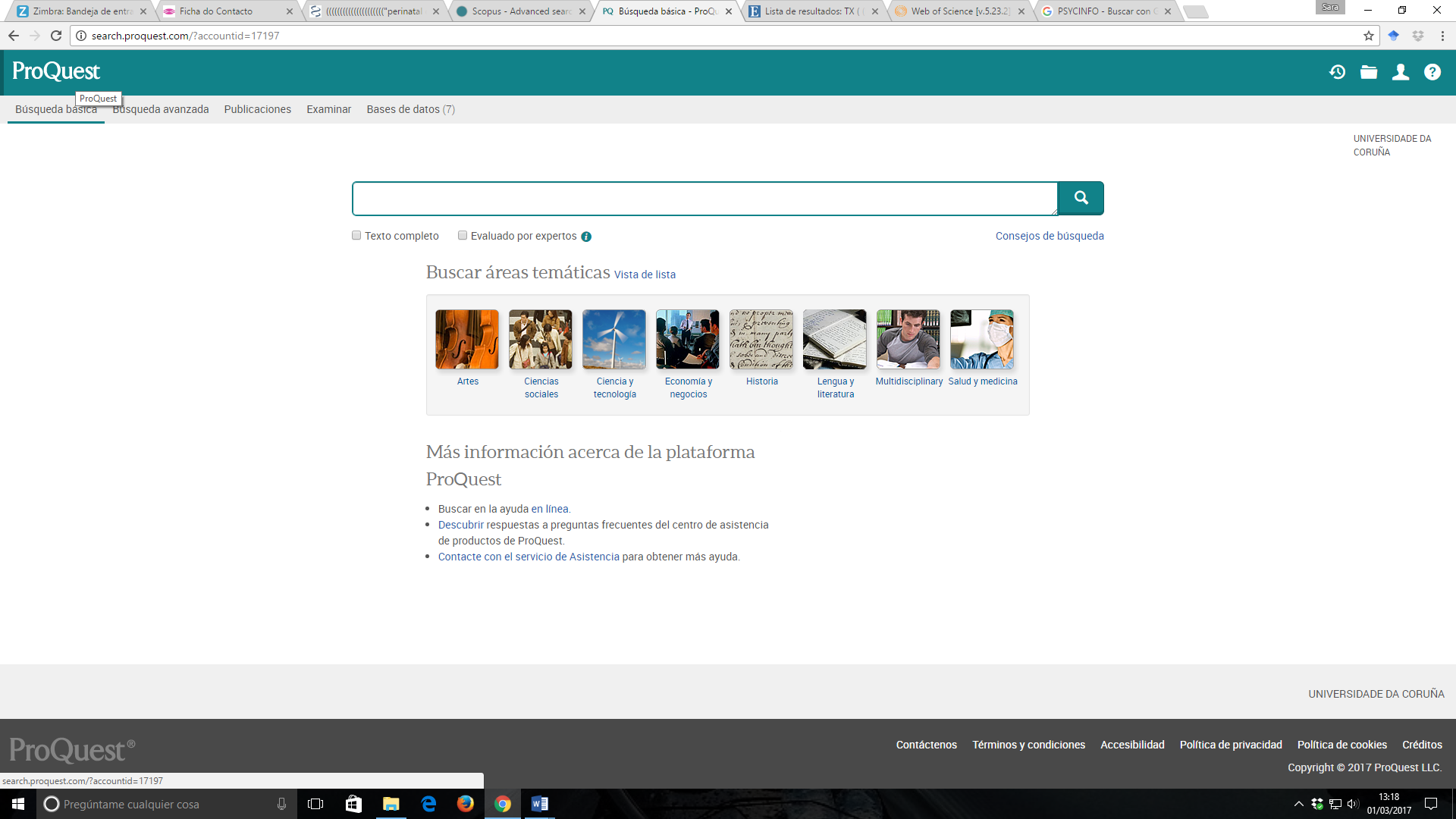 | PsycINFO |
| --- | --- |

| S1 | All fields: “experienc*” OR “view*” OR “opinion*” OR "Attitude*" OR "Emotion*" OR "Know*" OR “need*” OR "cope" OR "coping" OR "feeling" |
| --- | --- |
| S2 | All fields: "Nurs*" OR "first-line nurse*" OR "nursing care" OR "care" OR "caring" |
| S3 | All fields: "COVID-19" OR "Coronavirus" OR "coronavirus outbreak*" OR "COVID-19 Pandemic" OR (“Coronavirus disease”) |
| S4 | All fields: "Qualitative Research" OR "qualitative" OR "phenomenolog*" OR "grounded theory" OR "ethnograph*" OR "interview* " OR "focus group*" OR "narration" OR "narrative*" |
| S5 | S1 AND S2 |
| S6 | S3 AND S4 |
| S7 | S5 AND S6 |
| (“experienc*” OR “view*” OR “opinion*” OR "Attitude*" OR "Emotion*" OR "Know*" OR “need*” OR "cope" OR "coping" OR "feeling") AND ("Nurs*" OR "first-line nurse*" OR "nursing care" OR "care" OR "caring") AND ("COVID-19" OR "Coronavirus" OR "coronavirus outbreak*" OR "COVID-19 Pandemic" OR (“Coronavirus disease”)) AND ("Qualitative Research" OR "qualitative" OR "phenomenolog*" OR "grounded theory" OR "ethnograph*" OR "interview* " OR "focus group*" OR "narration" OR "narrative*") | |
| Language: English, Portuguese and Spanish. | |
| TOTAL: 79 | |

| 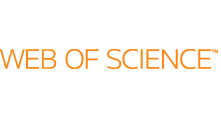 | Web of Science |
| --- | --- |

| S1 | TS=(“experienc*” OR “view*” OR “opinion*” OR "Attitude*" OR "Emotion*" OR "Know*" OR “need*” OR "cope" OR coping OR feeling*) |
| --- | --- |
| S2 | TS=("Nurs*" OR "first-line nurse*" OR "nursing care") |
| S3 | TS=("COVID-19" OR "Coronavirus" OR "coronavirus outbreak*" OR "COVID-19 Pandemic" OR “Coronavirus disease”) |
| S4 | TS=("Qualitative Research" OR qualitative OR phenomenolog* OR "grounded theory" OR ethnograph* OR interview* OR "focus group*" OR narration OR narrative*) |
| S5 | S1 AND S2 |
| S6 | S3 AND S4 |
| S7 | S5 AND S6 |
|  | **(“experienc*” OR “view*” OR “opinion*” OR "Attitude*" OR "Emotion*" OR "Know*" OR “need*” OR "cope" OR coping OR feeling*) AND ("Nurs*" OR "first-line nurse*" OR "nursing care") AND ("COVID-19" OR "Coronavirus" OR "coronavirus outbreak*" OR "COVID-19 Pandemic" OR “Coronavirus disease”) AND ("Qualitative Research" OR qualitative OR phenomenolog* OR "grounded theory" OR ethnograph* OR interview* OR "focus group*" OR narration OR narrative*)** |
| Language: English, Portuguese and Spanish. | |
| TOTAL: 205 | |
