# Supplementary material for: Walking a tightrope: A meta‐synthesis from frontline nurses during the COVID‐19 pandemic
Source: Nurs Inq. 2022 Apr 5:e12492. Online ahead of print. doi: 10.1111/nin.12492 (PMC9115365; doi:10.1111/nin.12492)
Supplement: Supplementary file 2 — Supporting information. [file NIN-9999-0-s001.docx]

**Studies assessed for meta-ethnography**

**(n= 15)**

Duplicate records excluded
(n = 338)

Records identified through database searching
(n = 803)

**Screening**

**Included**

**Eligibility**

**Identification**

Additional records identified through updated searching
(n = 1)

Records screened for duplicate
(n = 804)

Records screened

(Based on titles and abstract)
(n = 466)

Records excluded
(n = 419)

Records excluded, with reasons:

- Incorrect phenomenon of interest

(n= 11)

- Incorrect methodology

(n= 10)

- Incorrect sample

(n= 8)

- No primary article

(n= 3)

(n = 32)

Records assessed for eligibility

(Based on full text)
(n = 47)

Supplementary File 2 PRISMA flowchart
