# Supplementary material for: Walking a tightrope: A meta‐synthesis from frontline nurses during the COVID‐19 pandemic
Source: Nurs Inq. 2022 Apr 5:e12492. Online ahead of print. doi: 10.1111/nin.12492 (PMC9115365; doi:10.1111/nin.12492)
Supplement: Supplementary file 3 — Supporting information. [file NIN-9999-0-s005.docx]

**Supplementary File 3.** Quality assessment of included studies

| Articles | Questions | | | | | | | | | |
| --- | --- | --- | --- | --- | --- | --- | --- | --- | --- | --- |
|  | **1** | **2** | **3** | **4** | **5** | **6** | **7** | **8** | **9** | **10** |
| Andreu-Periz et al. (2020) | **✓** | **✓** | **✓** | **✓** | **✓** | **-** | **✓** | **✓** | **✓** | **✓** |
| Catania et al. (2020) | **✓** | **✓** | **✓** | **✓** | **✓** | **-** | **✓** | **✓** | **✓** | **✓** |
| Deliktas Demirci et al. (2020) | **✓** | **✓** | **✓** | [**✘**](https://es.wiktionary.org/w/index.php?title=%E2%9C%98&action=edit&redlink=1) | **✓** | [**✘**](https://es.wiktionary.org/w/index.php?title=%E2%9C%98&action=edit&redlink=1) | **✓** | **✓** | **✓** | **✓** |
| Galehdar et al. (2021) | **✓** | **✓** | **✓** | **✓** | **✓** | **-** | **✓** | **✓** | **✓** | **✓** |
| Galehdar et al. (2020) | **✓** | **✓** | **✓** | **✓** | **✓** | **-** | **✓** | **✓** | **✓** | **✓** |
| Iheduru-Anderson (2020) | **✓** | **✓** | **✓** | **✓** | **✓** | **-** | **✓** | **✓** | **✓** | **✓** |
| Jia (2020) | **✓** | **✓** | **✓** | **✓** | **✓** | **-** | **✓** | **✓** | **✓** | **✓** |
| Kackin et al., (2020) | **✓** | **✓** | **✓** | **✓** | **✓** | [**✘**](https://es.wiktionary.org/w/index.php?title=%E2%9C%98&action=edit&redlink=1) | **✓** | **✓** | **✓** | **✓** |
| Kalateh Sadati et al. (2020) | **✓** | **✓** | **✓** | **✓** | **✓** | - | **✓** | **✓** | **✓** | **✓** |
| Karimi et al. (2020) | **✓** | **✓** | **✓** | **✓** | **✓** | - | **✓** | **✓** | **✓** | **✓** |
| Lee and Lee (2020) | **✓** | **✓** | **✓** | **✓** | **✓** | **✓** | **✓** | **✓** | **✓** | **✓** |
| Liu et al. (2020) | **✓** | **✓** | **✓** | **✓** | **✓** | **-** | **✓** | **✓** | **✓** | [**✘**](https://es.wiktionary.org/w/index.php?title=%E2%9C%98&action=edit&redlink=1) |
| Schroeder et al. (2020) | **✓** | **✓** | **✓** | **✓** | **✓** | - | **✓** | **✓** | **✓** | [**✘**](https://es.wiktionary.org/w/index.php?title=%E2%9C%98&action=edit&redlink=1) |
| Sun et al. (2020) | **✓** | **✓** | **✓** | **✓** | **✓** | [**✘**](https://es.wiktionary.org/w/index.php?title=%E2%9C%98&action=edit&redlink=1) | **✓** | **✓** | **✓** | **✓** |
| Tan et al. (2020) | **✓** | **✓** | **✓** | **✓** | **✓** | - | **✓** | **✓** | **✓** | **✓** |

***Abbreviations: ✓*** *Yes* ***–*** *Unclear* ***🗶*** *No;* ***Critical appraisal questions****: 1) Was there a clear statement of the aims of the research? 2) Is the qualitative methodology appropriate? 3)* *Was the research design appropriate to address the aims of the research? 4) Was the recruitment strategy appropriate? 5) Were the data collected in a way that addressed the research issue? 6) Has the relationship between researcher and participants been adequately considered? 7) Have ethical issues been taken into consideration? 8) Was the data analysis sufficiently rigorous? 9) Is there a clear statement of findings? 10) How valuable is the research?*
