# Supplementary material for: Walking a tightrope: A meta‐synthesis from frontline nurses during the COVID‐19 pandemic
Source: Nurs Inq. 2022 Apr 5:e12492. Online ahead of print. doi: 10.1111/nin.12492 (PMC9115365; doi:10.1111/nin.12492)
Supplement: Supplementary file 4 — Supporting information. [file NIN-9999-0-s003.docx]

**Supplementary File 4** Characteristics of included studies

| Authors, (year), Location | Methods | Aim | Sample | Service | Data collection method | Key findings |
| --- | --- | --- | --- | --- | --- | --- |
| Andreu-Periz et al. (2020)  Spain | Qualitative phenomenological study | To deepen the knowledge about life experiences and support perceived by nurses who attended in-hospital patients with COVID-19 disease on haemodialysis during the months with the highest prevalence of the pandemic in Spain. | 10 nurses | Haemodialysis Unit | Semi-structured interviews | Four dimensions emerged from the speech analysis associated with different subcategories: ignorance about the disease, patient suffering, perceived support and professionals’ ability to cope |
| Catania et al. (2020)  Italy | A descriptive qualitative study | To explore nursing management issues within COVID-19 narratives of Italian front-line nurses. | 23 nurses | Acute care hospital settings: intensive care, emergency department, sub-intensive care and departments of infectious diseases | Testimonies | Six macro themes were identified as follows: organisational and logistic change; leadership models adopted to manage the emergency; changes in nursing approaches; personal protective equipment issues; physical and psychological impact on nurses; and team value/spirit. |
| Deliktas Demirci et al. (2020)  Turkey | Grounded theory | To explore the experiences and coping strategies of Turkish nurses working in pandemic units. | 15 nurses | COVID-19 pandemic units | In-depth interviews | A core category (‘It was difficult working in the unknown, but our struggle to touch lives gave us strength’) shows that all nurses felt heroic via the satisfaction of touching patients’ lives and uncertain. Four main categories emerged: being caught in the pandemic, empowerment for coping with the struggle, challenges during the coping process and effects of the pandemic on life. |
| Galehdar et al. (2021)  Iran | Qualitative research | To explore nurses' perception of taking care of patients with COVID-19. | 13 nurses | The department of inpatient care of COVID-19 patients | Semi-structured in-depth telephone interviews | Nurses experienced many challenges such as bad feeling of inefficiency, stress, excessive physical fatigue, dilemma between care delivery and pollution, and enclosed in protective equipment during taking care of patients with COVID-19, that all this can lead to decrease of the quality of patient care. However, the outbreak of COVID-19, along with difficulties and problems for the health staff, has also created opportunities for nurses and the nursing profession. Improving nurses' occupational status and morale and deepening the understanding of the nursing profession were among the benefits that nursing staff experienced during the outbreak of COVID-19. |
| Galehdar et al. (2020)  Iran | Qualitative research | To explore nurses’ experiences of psychological distress during care of patients with COVID-19 | 20 nurses | Emergency  Intensive care unit  Coronary care unit  General unit | Semi-structured in-depth telephone interviews | Data analysis revealed 11 categories including death anxiety, anxiety due to the nature of the disease, anxiety caused by corpse burial, fear of infecting the family, distress about time wasting, emotional distress of delivering bad news, fear of being contaminated, the emergence of obsessive thoughts, the bad feeling of wearing personal protective equipment, conflict between fear and conscience, and the public ignorance of preventive measures |
| Iheduru-Anderson (2020)  USA | Descriptive phenomenological design | To describe the lived experience of acute care nurses working with limited access to PPE during the COVID-19 pandemic | 28 nurses | Medical–surgical unit  Emergency department  Intensive care unit | Unstructured interviews | The major theme, emotional roller coaster, describes the varied intense emotions the nurses experienced during the early weeks of the pandemic, encompassing eight subthemes: scared and afraid, sense of isolation, anger, betrayal, overwhelmed and exhausted, grief, helpless and at a loss, and denial. Other themes include: self-care, ‘hoping for the best’, ‘nurses are not invincible’, and ‘I feel lucky’. The high levels of stress and mental assault resulting from the COVID-19 crisis call for early stress assessment of nurses and provision of psychological intervention to mitigate lasting psychological trauma. |
| Jia (2020)  China | Qualitative study | To examine the ethical challenges encountered by nurses caring for patients with COVID-19 and to provide nurses with suggestions and support regarding promotion of their mental health. | 18 nurses | Outpatient department  Internal medicine  Surgery  Emergency  Intensive care unit | Structured in-depth interviews | Nurses worked in a new environment to help COVID-19 patients. They stood on the frontline and fully devoted themselves to disease control regardless of any danger. However, at the same time, they had to cope with the ethical challenges brought by COVID-19. |
| Kackin et al., (2020)  Turkey | Descriptive phenomenological research | To determine the experiences and psychosocial problems of nurses caring for patients diagnosed with COVID-19 in Turkey. | 10 nurses | COVID-19 war department | Semi-Structured Interview Form | The theme of the effects of the outbreak was divided into working conditions, psychological effects and social effects; the theme of short-term coping strategies was divided into normalisation, refusal to dwell on experiences, avoidance, expression of emotions and distraction; and the theme of necessities was divided into psychosocial support and resource management. |
| Kalateh Sadati et al. (2020)  Iran | Qualitative study | To investigate nurses’ perceptions and experiences of COVID-19 outbreak in Iran | 24 nurses | Hospitals specified for COVID-19 treatment | Semi-structured interviews | The participants had faced a mysterious world created by the virus. No one had clear understanding of the new virus and knew how to tackle with such a virus. The main experiences were related to defected preparedness, the worst perceived risk, family protection, social stigma and sacrificial commitment. Urgent preparedness of facilities in such outbreaks is inevitable. Accordingly, psycho-social support of nurses and their families and strengthening their sacrificial commitments are proposed in these conditions. |
| Karimi et al. (2020)  Iran | A descriptive phenomenology | To explore the lived experiences of nurses caring for patients with COVID-19 in Iran. | 12 nurses | Coronavirus Centre | Semi-structured interviews | Mental condition, emotional condition, and care context were the main themes that emerged from the nurses' statements. Anxiety, stress, fear, witnessing the death of patients and colleagues, substandard care conditions, and a lack of facilities were the most pressing concerns identified in the nurses’ statements. |
| Lee and Lee (2020)  South Korea | A descriptive phenomenology | To explore the experiences of COVID-19-designated hospital nurses in South Korea who provided care for patients based on their lived experiences. | 18 nurses | COVID-19 isolation ward | In-depth interviews | The essential structure of the phenomenon was growth after the frontline battle against an infectious disease pandemic. Nine themes were identified: Pushed onto the Battlefield Without Any Preparation, Struggling on the Frontline, Altered Daily Life, Low Morale, Unexpectedly Long War, Ambivalence Toward Patients, Forces that Keep Me Going, Giving Meaning to My Work, and Taking Another Step in One’s Growth. The nurses who cared for patients with COVID-19 had both negative and positive experiences, including post-traumatic growth. |
| Liu et al. (2020)  China | Qualitative study | To explore the experiences of front-line nurses combating the coronavirus disease-2019 epidemic. | 15 nurses | General wards  Infectious disease wards  Intensive care units | Semi-structured in-depth interviews | Four theme categories emerged from the data analysis: (a) “Facing tremendous new challenges and danger”; (b) “Strong pressure because of fear of infection, exhaustion by heavy workloads and stress of nursing seriously ill COVID-19 patients”; (c) “Strong sense of duty and identity as a healthcare provider”; (d) “Rational understanding of the epidemic—the nurses believed that the epidemic would soon be overcome and would like to receive disaster rescue training.” |
| Schroeder et al. (2020)  USA | A qualitative descriptive study | To explore the experience of being a registered nurse caring for patients with COVID-19 at an urban academic medical centre during the early stages of the pandemic | 21 nurses | Emergency room, critical care, medical-surgical units, and float pool | In-person semi-structured interviews | Registered nurses perceived the clinical context as highly dynamic, but quickly adapted to pandemic-related care delivery. They felt a “sense of duty” to care for patients with COVID-19, despite being fearful of acquiring or spreading infection. Compared to clinical colleagues, registered nurses reported increased patient exposure and performed tasks previously assigned to other clinical team members. |
| Sun et al. (2020)  China | A phenomenological method | To explore the psychology of nurses caring for COVID-19 patients. | 20 nurses | Department of Infectious Diseases (negative pressure ward) | One-to-one interviews | First, negative emotions present in early stage consisting of fatigue, discomfort, and helplessness was caused by high-intensity work, fear and anxiety, and concern for patients and family members. Second, self-coping styles included psychological and life adjustment, altruistic acts, team support, and rational cognition. Third, we found growth under pressure, which included increased affection and gratefulness, development of professional responsibility, and self-reflection. Finally, we showed that positive emotions occurred simultaneously with negative emotions. |
| Tan et al. (2020)  China | A phenomenological method | To explore the work experience of clinical first-line nurses treating patients with coronavirus disease 2019 (COVID-19). | 30 nurses | Fever clinic, intensive care unit, emergency department, infection wards and isolation wards | semi-structured interviews | Two main categories were determined for the research: negative experiences during clinical first-line work and positive impacts of clinical first-line work. Under the first category, it was determined that nurses highlighted their psychological experiences with clinical first-line work and the material difficulties they faced. These difficulties mainly related to labour shortages and a lack of protective equipment and experience. |

*Abbreviations: United States of America (USA)*
