# Supplementary material for: Walking a tightrope: A meta‐synthesis from frontline nurses during the COVID‐19 pandemic
Source: Nurs Inq. 2022 Apr 5:e12492. Online ahead of print. doi: 10.1111/nin.12492 (PMC9115365; doi:10.1111/nin.12492)
Supplement: Supplementary file 5 — Supporting information. [file NIN-9999-0-s002.docx]

**Supplementary File 5** eMERGe reporting guideline

| **No.** | **Criteria Headings** | **Reporting Criteria** | **Meta-ethnography – criteria met (Page No)** |
| --- | --- | --- | --- |
| **Phase 1—Selecting meta-ethnography and getting started** | | | |
| *Introduction* | | |  |
| 1 | Rationale and context for the meta-ethnography | Describe the gap in research or knowledge to be filled by the meta-ethnography, and the wider context of the meta-ethnography | Pages 2-3 |
| 2 | Aim(s) of the meta-ethnography | Describe the meta-ethnography aim(s) | Page 3 |
| 3 | Focus of the meta-ethnography | Describe the meta-ethnography review question(s) (or objectives) | Page 3 |
| 4 | Rationale for using meta-ethnography | Explain why meta-ethnography was considered the most appropriate qualitative synthesis methodology | Page 3 |
| **Phase 2—Deciding what is relevant** | | |  |
| *Methods* | | |  |
| 5 | Search strategy | Describe the rationale for the literature search strategy | Page 4, Supplementary File 1 |
| 6 | Search processes | Describe how the literature searching was carried out and by whom | Page 4 |
| 7 | Selecting primary studies | Describe the process of study screening and selection, and who was involved | Page 4 |
| *Findings* | | |  |
| 8 | Outcome of study selection | Describe the results of study searches and screening | Page 6 |
| **Phase 3—Reading included studies** | | |  |
| *Methods* | | |  |
| 9 | Reading and data extraction approach | Describe the reading and data extraction method and processes | Page 5 |
| *Findings* | | |  |
| 10 | Presenting characteristics of included studies | Describe characteristics of the included studies | Page 6 |
| **Phase 4—Determining how studies are related** | | |  |
| *Methods* | | |  |
| 11 | Process for determining how studies are related | Describe the methods and processes for determining how the included studies are related: - Which aspects of studies were compared AND - How the studies were compared | Page 5 |
| *Findings* | | |  |
| 12 | Outcome of relating studies | Describe how studies relate to each other | Page 6 |
| **Phase 5—Translating studies into one another** | | |  |
| *Methods* | | |  |
| 13 | Process of translating studies | Describe the methods of translation**:** - Describe steps taken to preserve the context and meaning of the relationships between concepts within and across studies- Describe how the reciprocal and refutational translations were conducted- Describe how potential alternative interpretations or explanations were considered in the translations | Page 5 |
| *Findings* | | |  |
| 14 | Outcome of translation | Describe the interpretive findings of the translation. | Page 6 |
| **Phase 6—Synthesizing translations** | | |  |
| *Methods* | | |  |
| 15 | Synthesis process | Describe the methods used to develop overarching concepts (“synthesised translations”) Describe how potential alternative interpretations or explanations were considered in the synthesis | Page 5 |
| *Findings* | | |  |
| 16 | Outcome of synthesis process | Describe the new theory, conceptual framework, model, configuration, or interpretation of data developed from the synthesis | Pages 6-12 and figure 2 |
| **Phase 7—Expressing the synthesis** | | |  |
| *Discussion* | | |  |
| 17 | Summary of findings | Summarize the main interpretive findings of the translation and synthesis and compare them to existing literature | Pages 12-15 |
| 18 | Strengths, limitations, and reflexivity | Reflect on and describe the strengths and limitations of the synthesis: - Methodological aspects—for example, describe how the synthesis findings were influenced by the nature of the included studies and how the meta-ethnography was conducted.- Reflexivity—for example, the impact of the research team on the synthesis findings | Page 15 |
| 19 | Recommendations and conclusions | Describe the implications of the synthesis | Pages 12-16 |
